# Letter to the editor: Some reflections on ‘Endonasal dacryocystorhinostomy in children: Our experience’

**DOI:** 10.1097/JS9.0000000000001509

**Published:** 2024-05-03

**Authors:** Zijian Chen, Shizhou Cheng, Zuhai Zhang

**Affiliations:** Department of Ophthalmology, The First Affiliated Hospital of Yangtze University, Jingzhou, Hubei Province, People’s Republic of China

*Dear Editor,*

We read the article ‘Endonasal dacryocystorhinostomy in children: Our experience’^1^ with interest. We have some thoughts of our own.

First of all, neonatal lacrimal duct obstruction can typically heal in about 6 weeks under normal circumstances. If it does not heal, we can assist in opening the lower end of the nasolacrimal duct through local massage and other conservative treatments. If conservative treatment is not effective, after 6 months, we can consider tear duct exploration and nasolacrimal intubation. Endoscopic dacryocystorhinostomy (DCR) is a more invasive high anesthesia risk, especially for infants and young children, usually when the tear duct exploration fails, or the child is diagnosed with acute and chronic lacrimal dacryocystitis^2^.

Placement of a lacrimal stent in endoscopic DCR is the most common method to prevent the closure of a nasolacrimal stoma. The advantages of lacrimal stent are that they can delay the fibrous closure of the anastomotic tissue at the lacrimal sac during postoperative healing, maintain anastomotic patency, and improve the surgical outcome of endoscopic DCR, but the disadvantages are that the use of a lacrimal stent in endoscopic DCR and prolonged implantation may cause granulation of the tissue, which is susceptible to postoperative infections, adhesions, and tearing of the tear point^3^ The duration reported in the relevant literature ranges from 4 weeks to 6 months. The optimal duration of silicone drain placement is therefore controversial, both to ensure that the desired surgical outcome is achieved and that there is a reduction in associated postoperative complications.

The mucosa of the operative cavity enters an inflammatory phase within 3–10 weeks after surgery. During this period, vesicles, polyps, and granulation tissue in the operative cavity begin to grow, fibrous connective tissue proliferates and adheres, and mucosal regeneration and epithelialization occur simultaneously. Improper handling of diseased tissue can lead to adhesions in the operative cavity, narrowing of the operative and sinus cavities, and even occlusion^4^. The lacrimal stent is placed as a foreign body in the anastomosis, and the risk of anastomotic and lacrimal granulation tissue formation, as well as infection, increases progressively with time. Moreover, experiments in mice have shown that subepithelial thickness index (SFI) and epithelial thickness index (ETI) values increase significantly at 2 weeks^5^, and the mucosal healing rate will be greater in toddlers and children than in adults. Therefore, we believe that removal of the lacrimal stent at 3 months postoperatively increases complications such as anastomosis and lacrimal granulation, postoperative infections, and adhesions, resulting in a decreased success rate. We should shorten its placement time.

Moreover, we have a slight concern about the topical use of mitomycin C. Mitomycin C may inhibit fibrosis and blood vessel growth by inhibiting the synthesis of DNA, RNA, and proteins in fast-growing cells, thereby decreasing the likelihood of anastomotic stenosis. However, strong evidence of mitomycin C’s effect still does not exist, and we believe that whether mitomycin C can act accurately on the anastomosis each time will directly affect the results of the experiment. Moreover, mitomycin C will inevitably act on the damaged mucosal tissue, which will affect the healing of the damaged tissue.

## Ethics approval and consent to participate

Ethics approval and consent to participate are not applicable to this letter.

## Consent for publication

Consent for publication from participants is not applicable to this study.

## Sources of funding

No relevant funding was received for this study.

## Author contribution

Z.C.: writing the paper; S.C.: study concept; and Z.Z.: study design.

## Conflicts of interest disclosure

The authors declare no conflicts of interest.

## Research registration unique identifying number (UIN)

No human experimentation is involved.

## Guarantor

Zuhai Zhang.

## Data availability statement

This letter is a discussion of the previous article and therefore does not contain a data statement.

## Provenance and peer review

Not commissioned, externally peer-reviewed.
